# Validation and adaptation of an instrument for assessing the perceived organizational infection prevention climate: evidence from Chinese healthcare workers

**DOI:** 10.1017/ash.2025.114

**Published:** 2025-09-03

**Authors:** Yanfang Huang

**Affiliations:** Department of Critical Care Medicine, The Affiliated HospitalSouthwest Medical University, China

**Keywords:** Infection prevention climate, Questionnaire, Healthcare workers, Reliability, Validity

## Abstract

In recent years, organizational factors such as infection prevention climate have been recognized as important factors of healthcare workers’ adherence to infection prevention practices. However, there is a lack of instruments with good reliability and validity to measure infection prevention climate within organizations in Chinese context. Therefore, this study aims to translate, culturally adaptation and test for the psychometric properties of the Chinese version of Leading a Culture of Quality for Infection Prevention (CLCQ-IP). The original scale was translated into Chinese through 1) Forward translation; 2) Expert review; 3) Back translation; 4) Applicability evaluation. Then, a multicenter cross-sectional survey was conducted using the CLCQ-IP. Reliability in terms of internal consistency was evaluated. The content validity, exploratory factor analysis, confirmatory factor analysis, were tested for assessing the construct validity of the CLCQ-IP. After linguistic and cultural adaptation, the CLCQ-IP was finally formed with 19 items in 4 dimensions and a total 882 HCWs from 4 provinces finished the survey. The overall Cronbach’s alpha of the CLCQ-IP was 0.865. The items of content validity index, ICVI of the C-SPQ ranged from 0.875 to 1.00, and the scale of content validity index S-CVI/AVE was 0.894. In terms of construct validity, the exploratory factor analysis extracted a total of 4 factors, which were consistent with the original scale. The factor loadings of each item were above 0.70, and the cumulative variance contribution to the scale was 71.88 %. The Confirmatory factor analysis showed the good model indicators: *x*^"^/*df* =1.508, RMSEA=0.41, GFI=0.934, AGFI=0.912, NFI=0.953, TLI=0.981, CFI=0.984. The results of the study show empirical evidence of validity and reliability of CLCQ-IP can be highly recommended to be widely used among Chinses HCWs.

